# Fluorine‐Driven Enhancement of Birefringence in the Fluorooxosulfate: A Deep Evaluation from a Joint Experimental and Computational Study

**DOI:** 10.1002/advs.202003594

**Published:** 2021-06-04

**Authors:** Wenqi Jin, Wenyao Zhang, Abudukadi Tudi, Liying Wang, Xin Zhou, Zhihua Yang, Shilie Pan

**Affiliations:** ^1^ CAS Key Laboratory of Functional Materials and Devices for Special Environments Xinjiang Technical Institute of Physics & Chemistry of CAS Xinjiang Key Laboratory of Electronic Information Materials and Devices 40‐1 South Beijing Road Urumqi 830011 China; ^2^ Center of Materials Science and Optoelectronics Engineering University of Chinese Academy of Sciences Beijing 100049 China; ^3^ State Key Laboratory of Magnetic Resonance and Atomic and Molecular Physics National Center for Magnetic Resonance in Wuhan Wuhan Institute of Physics and Mathematics Innovation Academy for Precision Measurement Science and Technology Chinese Academy of Sciences Wuhan 430071 China

**Keywords:** birefringence, deep‐ultraviolet, electronic structure, fluorooxosulfate, functional module

## Abstract

Understanding and exploring the functional modules (FMs) consisting of local atomic groups can promote the development of the materials with functional performances. Oxygen‐containing tetrahedral modules are popular in deep‐ultraviolet (DUV) optical materials, but their weak optical anisotropy is adverse to birefringence. Here, the fluorooxosulfate group is proved as a new birefringence‐enhanced FM for the first time. The birefringence of fluorooxosulfates can be 4.8–15.5 times that of sulfates with the same metal cations while maintaining a DUV band gap. The polarizing microscope measurement confirms the birefringence enhancement by using the millimeter crystals experimentally. The theoretical studies from micro and macro levels further reveal a novel universal strategy that the fluorine induced anisotropic electronic distribution in fluorooxo–tetrahedral group is responsible for the enhancement of birefringence. This study will guide the future discovery of DUV optical materials with enlarged birefringence.

## Graphical abstract

A novel design and synthesis strategy for birefringent materials. The fluorooxosulfate group is proved as a new birefringence‐enhanced FM for the first time. The fluorine‐free tetrahedra substituted by the fluorooxo‐tetrahedral group can enhance birefringence while maintaining short cut‐off edge, which is proved by the experiments. This study provides a feasible way to design DUV optical materials with enlarged birefringence.

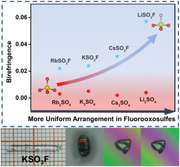

## 1 Introduction

Capturing the functional modules (FMs) that can mainly determine a compound's key properties is crucial to the design and prediction of functional materials with high performance.^[^ ^1^ ^]^ Birefringence is a key factor to modulate the polarization of light or phase‐matching in birefringent or nonlinear optical (NLO) materials,^[^ ^2^ ^]^ which have significant applications in material‐processing, laser micromachining, photolithography, optical measurements, and manipulating entangled photons.^[^ ^3^ ^]^ Learning from the known birefringent or NLO materials, many excellent materials contain (CO_3_)^2−^, (BO_3_)^3−^ or (B_3_O_6_)^3−^,^[^ ^4^ ^]^ such as CaCO_3_,^[^ ^5^ ^]^ *α*/*β*‐BaB_2_O_4_ (*α*/*β*‐BBO),^[^ ^4^, ^6^ ^]^ and KBe_2_BO_3_F_2_ (KBBF).^[^ ^7^ ^]^ In view of their microscopic structures, the common characteristics are the planar structures, which are regarded as the birefringence‐enhanced FMs. However, the nonbonding orbitals in non‐condensed (B_3_O_6_)^3−^, for example, may reduce the band gap which is not expected in deep‐ultraviolet (DUV) region.^[^ ^4^, ^8^ ^]^ Therefore, to explore new birefringence‐enhanced FMs that can realize the enhanced birefringence while maintaining DUV band gap has become one of the hotspots.

In general, the primary factors that influence the birefringence of a crystal are anionic framework and metal cation polyhedra.^[^ ^9^ ^]^ Currently, some strategies have been tried to explore new birefringence‐enhanced FMs. Introducing metal cation with stereochemical activity lone pair (SCALP), *d* ^0^ transition metal with the second‐order Jahn–Teller effect or *d* ^10^ transition metal can enhance the birefringence, which has been proven in experiment.^[^ ^10^ ^]^ Like Rb_3_PbBi(P_2_O_7_)_2_,^[^ ^11^ ^]^ Sn_2_B_5_O_9_Cl,^[^ ^9^ ^]^ BaSn_2_(PO_4_)_2,_^[^ ^12^ ^]^ and LiHgPO_4_,^[^ ^13^ ^]^ their birefringence gets great enhancement in comparison with the corresponding isostructural alkaline‐earth compounds. The Pb^2+^ and Sn^2+^ cations with SCALP or Hg^2+^ cation with high polarizability make a large contribution to the birefringence due to the polarizability anisotropy of distorted metal polyhedra. In addition to the common birefringence‐enhanced FMs (CO_3_)^2−^, (BO_3_)^3−^ or (B_3_O_6_)^3−^, choosing novel planar structures including (C_3_N_3_O_3_)^3−^, (B_2_O_5_)^4−^ and (BO_2_)_∞_^−^ chain is also one effective way owing to their large anisotropy of polarizabilities.^[^ ^14^ ^]^ Accordingly, KLi(HC_3_N_3_O_3_)·2H_2_O,^[^ ^15^ ^]^ Li_2_Na_2_B_2_O_5_^[^ ^14e^ ^]^, and Ca(BO_2_)_2_^[^ ^14f^ ^]^ exhibit large birefringence and have been regarded as promising birefringent materials. Oxygen‐containing tetrahedral groups such as (BO_4_)^5−^, (PO_4_)^3−^, and (SO_4_)^2−^ are usually popular in UV and DUV optical materials owing to their DUV transparent superiority. However, they are rarely favored for birefringence due to their undesirably weakly optical anisotropy. BPO_4_, for example, has a small birefringence ≈0.005 @1064 nm, which hinders its phase‐matching ability.^[^ ^16^ ^]^ Recently, fluorooxoborates have been widely concerned as promising birefringent/NLO materials in the DUV region.^[^ ^17^ ^]^ Typical fluorooxoborates include M_2_B_6_O_9_F_2_ (M = Li, Na, Na_0.5_/Rb_0.5_),^[^ ^18^ ^]^ AB_4_O_6_F (A = NH_4_, Na, Rb, Cs, K_0.5_/Cs_0.5_, Rb_0.5_/Cs_0.5_),^[^ ^19^ ^]^ MB_5_O_7_F_3_ (M = Ca, Sr),^[^ ^20^ ^]^ and MB_4_O_6_F_2_ (M = Sr, Ca, Ba).^[^ ^21^ ^]^ The O atoms in (BO_4_)^5−^ are substituted by the F atoms with a large electronegativity, forming the [BOF] ((BO*_x_* F_4−_ *_x_*) ^(^ *^x^* ^+1)−^, *x* = 1, 2, 3) groups, which exhibit a superiority in the UV cutoff edge and the second harmonic generation (SHG) response.^[^ ^17^, ^22^ ^]^ The [BOF] groups with the combination of (BO_3_)^3−^ groups also show benefits in optical anisotropy. Following the [BOF] FMs, the [POF] ((PO*_x_* F_4−_ *_x_*)^(^ *^x−^* ^1)−^, *x*= 1, 2) groups have also attracted attention; consequently, several fluorophosphates including (NH_4_)_2_PO_3_F and NaNH_4_PO_3_F·H_2_O have been reported for their benign optical properties.^[^ ^23^ ^]^ Clarifying the origin of enhanced birefringence is of great importance to design birefringent/NLO materials.^[^ ^24^ ^]^ To date, there is no systematic research to unveil the origin of fluorooxo‐tetrahedral groups in enhancing birefringence as compared to fluorine‐free tetrahedral groups (i.e., (BO_4_)^5−^, (PO_4_)^3−^, and (SO_4_)^2−^). In addition, whether the enhancement is universal to other systems remains unclear. And, are there any other new birefringence‐enhanced FMs?

Aiming at the design of birefringence‐enhanced FMs, a material design strategy that could enhance birefringence and simultaneously keep the short cutoff edge is expected to be proposed. Here, besides (PO_3_F)^2−^, (SO_3_F)^−^, an anionic group similar to [BOF] and [POF] but uninvestigated in its optical anisotropy, captured our attention as well. We studied systemically the fluorooxosulfates and fluorophosphates with the fluorooxo‐tetrahedral groups. The difference in polarizability anisotropy between the fluorine‐free tetrahedral groups ((SO_4_)^2−^, (PO_4_)^3−^, [MO_4_] (M = S, P) for short) and the fluorooxo‐tetrahedral groups ((SO_3_F)^−^, (PO_3_F)^2−^, [MO_3_F] (M = S, P) for short) was analyzed with the combination of the first principles calculation. The optical properties of the fluorophosphates and fluorooxosulfates were calculated and analyzed in comparison with the orthophosphates and sulfates, namely, Na_3_PO_4_,^[^ ^25^ ^]^ Na_2_PO_3_F,^[^ ^26^ ^]^ Li_2_SO_4,_^[^ ^27^ ^]^ and LiSO_3_F,^[^ ^28^ ^]^ and the response electronic distribution anisotropy (REDA) method was employed to discuss the bonding behavior of different groups.^[^ ^29^ ^]^ K_2_SO_4_,^[^ ^30^ ^]^ KSO_3_F,^[^ ^31^ ^]^ Rb_2_SO_4_,^[^ ^32^ ^]^ RbSO_3_F,^[^ ^33^ ^]^ Cs_2_SO_4_,^[^ ^34^ ^]^ CsSO_3_F,^[^ ^33^ ^]^ (NH_4_)_2_SO_4_,^[^ ^35^ ^]^ and NH_4_SO_3_F^[^ ^36^ ^]^ were calculated for optical properties as well. In additon, K_2_SO_4_ and KSO_3_F, (NH_4_)_2_SO_4_ and NH_4_SO_3_F were synthesized and the crystals were grown to verify the accuracy of the calculated properties. To clarify the enhancement mechanism in the optical anisotropy, the electronic distributions of [MO_3_F] (M = S, P) groups were analyzed in comparison with [MO_4_] (M = S, P) groups. Consequently, new birefringent‐enhanced FMs were screened and a novel universal strategy was proposed for the enhancement of the birefringence.

## 2 Results and Discussion

**Figure 1:**
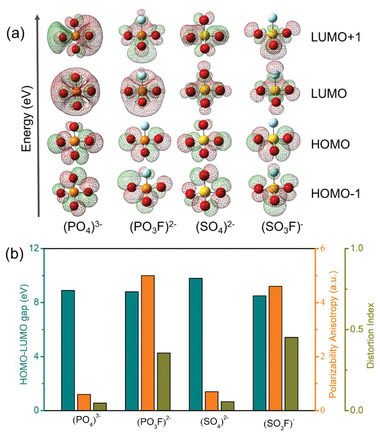
a) Frontier molecular orbital distributions for the (PO_4_)^3−^, (PO_3_F)^2−^, (SO_4_)^2−^, and (SO_3_F)^−^ anionic groups. The red, orange, cyan, and yellow balls represent the oxygen, phosphorus, fluorine, and sulfur atoms, respectively. b) Comparison of the HOMO–LUMO gap, polarizability anisotropy, and distortion index for the (PO_4_)^3−^, (PO_3_F)^2−^, (SO_4_)^2−^, and (SO_3_F)^−^ anionic groups.

To reveal the birefringence‐related functions of the (PO_4_)^3−^, (PO_3_F)^2−^, (SO_4_)^2−^, and (SO_3_F)^−^ anionic groups, we investigated their geometrical and electronic structures, which are extracted from the corresponding primitive cells, at the molecular level. First, the introduction of fluorine leads to the apparent structural distortion of the anionic groups, the calculated distortion index from the (PO_4_)^3−^ to (PO_3_F)^2−^ is 0.047 and 0.355, from (SO_4_)^2−^ to (SO_3_F)^−^ is 0.055 and 0.452, respectively (**Figure** **1**). As is well‐known to all, optical property is closely related to the characteristic occupied and the unoccupied states near the Fermi level. Therefore, the orbital spatial distributions of the highest occupied molecular orbital (HOMO), HOMO‐1, the lowest unoccupied molecular orbital (LUMO) and LUMO+1 for these anionic groups are shown in Figure 1a. It is obvious that the electron distributions of the (PO_4_)^3−^ and (SO_4_)^2−^ units are relatively uniform owing to their symmetrical structures, with a HOMO–LUMO gap of 8.9 and 9.8 eV, respectively. For the (PO_3_F)^2−^ and (SO_3_F)^−^, with a HOMO–LUMO gap of 8.8 and 8.5 eV, respectively, we can find that HOMO and HOMO‐1 are occupied by the nonbonding 2*p* orbitals of the O atom while the F atom is barely involved due to its large electronegativity. LUMO and LUMO+1 are constituted by the anti‐*σ* P─O/F and S─O/F bonds. Hence, the electronic cloud distributions of the (PO_3_F)^2−^ and (SO_3_F)^−^ show relatively asymmetrical distributions due to their asymmetrical structures which lead to different anisotropy of polarizability compared to those of the (PO_4_)^3−^ and (SO_4_)^2−^ units.

The calculated polarizability anisotropy of the (PO_4_)^3−^, (PO_3_F)^2−^, (SO_4_)^2−^, and (SO_3_F)^−^ units is 0.6, 5.0, 0.7, and 4.6, respectively (Figure 1b). The theoretical estimation of the polarizability anisotropy for anionic groups is in good agreement with the results obtained from the distributions of frontier molecular orbitals. What's more, the calculated HOMO–LUMO gaps of the (PO_3_F)^2−^ and (SO_3_F)^−^ groups are close to each other and correspond to extremely short UV cutoff edges. These calculated results suggest that the flurooxo‐tetrahedral groups can make a larger polarizability anisotropy, as well as maintain large HOMO–LUMO gap. From above analysis, the asymmetrical structures of the [MO_3_F] (M = S, P) groups possess larger polarizability anisotropy, and maintain wide HOMO–LUMO gaps, which indicate further that the fluorooxo‐tetrahetral groups offset the shortplank problem of the (PO_4_)^3−^ and (SO_4_)^2−^ units.

**Figure 2:**
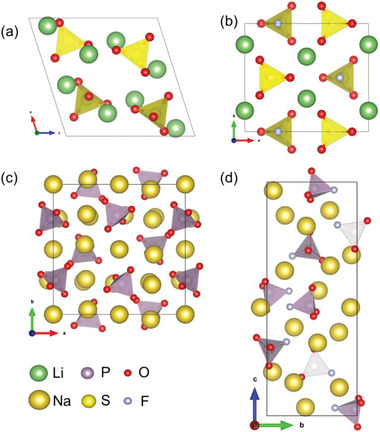
Crystal structures of a) Li_2_SO_4_, b) LiSO_3_F, c) Na_3_PO_4,_ and d) Na_2_PO_3_F.

To further check the availability of the [MO_3_F] (M = S, P) groups improving birefringence performance, we investigated the structure–properties relationship. There are 20 structures of fluorooxosulfates ((SO_3_F)^−^ as the only anionic group, and the number of elements is 4 or 5) in the inorganic crystal structure database (ICSD, 2021‐1, version 4.5.0, by Fachinformationszentrum Karlsruhe, Germany), in which we selected LiSO_3_F as representative for theoretical analysis due to its alkali metal cation and structure without disorder. Accordingly, the sulfate Li_2_SO_4_ was chosen as a comparison. For the orthophosphate and fluorophosphate, Na_3_PO_4_ and Na_2_PO_3_F were selected. The structures of Li_2_SO_4_, LiSO_3_F, Na_3_PO_4_, and Na_2_PO_3_F, optimized by CASTEP, are shown in **Figure** **2**. Among them, Na_3_PO_4_ and Na_2_PO_3_F are non‐centrosymmetric and crystallize in space group *P* $\bar{4}$2_1_ *c* and *P*2_1_2_1_2_1_, respectively. Li_2_SO_4_ and LiSO_3_F crystallize in centro‐symmetric space groups *P*2_1_ */a* and *C*2*/m*, respectively. As shown in Figure 2, the crystal structures of Na_3_PO_4_ and Li_2_SO_4_ are composed by the fluorine‐free tetrahedra (PO_4_)^3−^ and (SO_4_)^2−^, respectively. Na_2_PO_3_F and LiSO_3_F are composed of fluorooxo‐tetrahedral groups (PO_3_F)^2−^ and (SO_3_F)^−^, respectively. Notably, the arrangement of anionic groups in Li_2_SO_4_ and LiSO_3_F is ordered (for example, the directions of S─F bonds in the (SO_3_F)^−^ tetrahedra are along the *c* axis although half of them are arranged in the opposite direction) while in Na_3_PO_4_ and Na_2_PO_3_F, they are more random. K_2_SO_4_, KSO_3_F, Rb_2_SO_4_, RbSO_3_F, Cs_2_SO_4_, CsSO_3_F, (NH_4_)_2_SO_4_, and NH_4_SO_3_F were calculated as well, and their structures were presented in Figure S1, Supporting Information.

**Figure 3:**
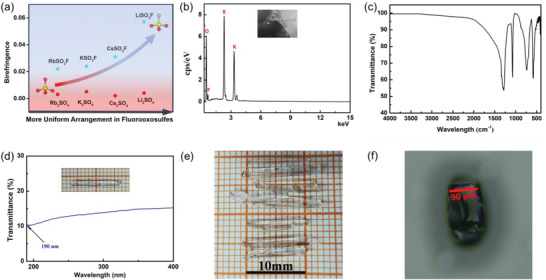
a) Calculated birefringences for alkali metal sulfates versus alkali metal fluorooxosulfates. b) The energy dispersive X‐ray (EDX) spectroscopy of KSO_3_F. c) The IR spectrum of KSO_3_F. d) The transmission spectrum of KSO_3_F from 190 to 400 nm. e) The millimeter‐scale crystals of KSO_3_F grown by hydrothermal reaction. f) The thickness of KSO_3_F crystal for polarizing microscope measurement.

In view of the birefringences calculated by the first‐principles methods, the title compounds Na_3_PO_4_, Na_2_PO_3_F, Li_2_SO_4_, and LiSO_3_F exhibit hierarchical phenomena, as shown in **Table** **1**. Na_3_PO_4_ has a very small birefringence ≈0.004 at 546 nm. Interestingly, the birefringence of Na_2_PO_3_F exhibits an apparent enhancement ≈0.022 at 546 nm, which is consistent with the previous study.^[^ ^23a^ ^]^ The similar situation can also be seen in Li_2_SO_4_ and the fluorooxosulfate LiSO_3_F; the birefringence is 0.004 at 546 nm for Li_2_SO_4_ and 0.057 at 546 nm for LiSO_3_F. More surprisingly, the fluorooxosulfate exhibits a much higher enhancement in birefringence from Li_2_SO_4_ to LiSO_3_F than that from Na_3_PO_4_ to Na_2_PO_3_F. Furthermore, Na_2_PO_3_F and LiSO_3_F have DUV cutoff edges (6.21 and 7.68 eV, respectively) and possess relatively large birefringences (Table 1). It is demonstrated that the birefringences of Na_2_PO_3_F and LiSO_3_F get greatly enhanced while their band gaps almost remain unchanged after introducing F atom into Na_3_PO_4_ and Li_2_SO_4_. These results mean that Na_2_PO_3_F and LiSO_3_F can achieve the balance between enhanced birefringence and band gap. These results confirm the microscopic analysis that the flurooxo‐tetrahedral groups [MO_3_F] (M = S, P) exhibit much larger polarizability anisotropy than that of the corresponding fluorine‐free tetrahedral groups [MO_4_] (M = S, P). The same phenomena can be seen in K_2_SO_4_ (or Rb_2_SO_4_, Cs_2_SO_4_, (NH_4_)_2_SO_4_) and KSO_3_F (or RbSO_3_F, CsSO_3_F, NH_4_SO_3_F) (Table 1 and **Figure** **3**a).

**Table 1:** Calcluated band gap and birefringence as well as experimental results

	*E*_g_ [eV]			*∆*n ^a)^	
Crystals	GGA	HSE06	Exp.	Cal.	Exp.	Enhancement of cal. ∆*n*
Na_3_PO_4_	3.63	5.86	—	0.004	—	0.019
Na_2_PO_3_F	4.19	6.21	—	0.023	—	
Li_2_SO_4_	6.04	7.86	—	0.004	—	0.053
LiSO_3_F	5.82	7.68	—	0.057	—	
K_2_SO_4_	4.84	7.29	>6.5	0.005	≥0.002	0.019
KSO_3_F (model I)	5.06	6.86	>6.5	0.024	≥0.019	
Rb_2_SO_4_	4.75	6.68	—	0.003	—	0.019
RbSO_3_F (model I)	5.06	6.75	—	0.022	—	
Cs_2_SO_4_	4.89	6.61	—	0.002	—	0.029
CsSO_3_F	5.16	6.75	—	0.031	—	
(NH_4_)_2_SO_4_	4.90	6.82	>6.5	0.020	≥0.011	0.024
NH_4_SO_3_F	5.02	6.70	—	0.044	≥0.018	

^a)^ The calculated and experimental birefringences at 546 nm.

To show that the introduction of the F atom in fluorine‐free groups can increase the birefringence, we also employed REDA method to calculate the bonding electron density difference (Δ*ρ* ^b^) of anionic groups in Na_3_PO_4_, Na_2_PO_3_F, Li_2_SO_4_, and LiSO_3_F. As shown in **Table** **2**, the (PO_3_F)^2−^ and (SO_3_F)^−^ units have significantly larger Δ*ρ* ^b^ values than those of the (PO_4_)^3−^ and (SO_4_)^2−^ units. And the enhancement in Δ*ρ* ^b^ from (PO_4_)^3−^ to (PO_3_F)^2−^ is smaller than that from (SO_4_)^2−^ to (SO_3_F)^−^. These results are generally in agreement with the increase of macro birefringences. However, the increase of polarizability anisotropy from (PO_4_)^3−^ to (PO_3_F)^2−^ is larger than that from (SO_4_)^2−^ to (SO_3_F)^−^ (Figure 1b), which is not accordant with the calculated Δ*ρ* ^b^ values and macro birefringences. The main reason is that the Δ*ρ* ^b^ value obtained by the REDA method contains the arrangement of anionic groups, and the arrangements of anionic groups in Li_2_SO_4_ and LiSO_3_F are aligned while those in Na_3_PO_4_ and Na_2_PO_3_F are random (Figure 2). As is well known, the parallel or antiparallel arrangement is beneficial to the superposition of micro polarizability anisotropy, like CaCO_3_^[^ ^5^ ^]^ and *γ*‐Be_2_BO_3_F_2_.^[^ ^37^ ^]^ In short, small bonding electron density difference in fluorine‐free groups can be tuned by introducing the F atom into tetrahedral modules, which will benefit the enhancement in birefringence.

**Table 2:** Bonding electron density difference (*Δρ*b) for anionic groups in compounds calculated by the REDA method

Compounds	Groups	Δ*ρ* ^b^ (× 10^−3^)
Na_3_PO_4_	(PO_4_)^3−^	0.2
Na_2_PO_3_F	(PO_3_F)^2−^	7.4
Li_2_SO_4_	(SO_4_)^2−^	0.6
LiSO_3_F	(SO_3_F)^−^	11.1

To verify the calculated results, we grew the crystal of KSO_3_F successfully with dimensions of 10 mm × 1 mm × 1 mm using hydrothermal method and fully characterized (Figure 3). The pure phases of KSO_3_F were synthesized and checked by powder X‐ray diffraction (Figure S2, Supporting Information). Single crystal diffraction data were collected at 296 and 150 K and the structure data are listed in Table S1–S3, Supporting Information. The elemental analysis by energy dispersive X‐ray (EDX) spectroscopy for KSO_3_F verifies the validity of the F element in the structure (Figure 3b). The infrared (IR) spectrum of KSO_3_F further confirms the presence of the O─S─F formation vibration (the peak at 570 cm^‐1^) and S─F stretching vibration (the peak at 750 cm^−1^) of the (SO_3_F)^−^ groups (Figure 3c).^[^ ^38^ ^]^ The solid state nuclear magnetic resonance (NMR) spectroscopy was also applied to further confirm the existence of the F element (See Figure S3 and more details, Supporting Information.). The transmission spectrum of KSO_3_F crystalline sample from 190 to 400 nm is shown in Figure 3d. The results show that the DUV cutoff edge of KSO_3_F is less than 190 nm. This confirms our predicted values above and indicates that the crystal can be used in the DUV region.

The birefringence of KSO_3_F was measured on a ZEISS Axio Scope (A1 polarizing microscope).^[^ ^39^ ^]^ The retardation value of a KSO_3_F crystal with a crystal thickness of 90 µm is 1.6937 µm (Figure 3f; and Figure S4, Supporting Information). Based on the formula (Equation (1) in the Experimental Section), the refractive index difference of the as‐measured crystal is about 0.019 at the wavelength of 546 nm. Notably, the plane of the measured crystal may not be parallel to the optical axis or the optical axis plane, so that the real birefringence should be equal or larger than 0.019, which is almost consistent with the calculated birefringence of KSO_3_F (model I) (Figure S5, Supporting Information). The polarizing microscope measurement indicates that KSO_3_F has a relatively large birefringence in DUV region. In addition, the crystals of NH_4_SO_3_F, K_2_SO_4_, and (NH_4_)_2_SO_4_ were grown with dimensions of 5 mm × 1 mm × 1 mm, 3 mm × 3 mm × 1 mm, and 3 mm × 3 mm × 1 mm, respectively. The transmission spectrum and polarizing microscope measurement for NH_4_SO_3_F, K_2_SO_4_, and (NH_4_)_2_SO_4_ were performed to verify our calculated results (Figures S7–S10, Supporting Information); the experimental data are also listed in Table 1. These results support the proposal that the fluorooxosulfate group is a birefringence‐enhanced FM.

**Figure 4:**
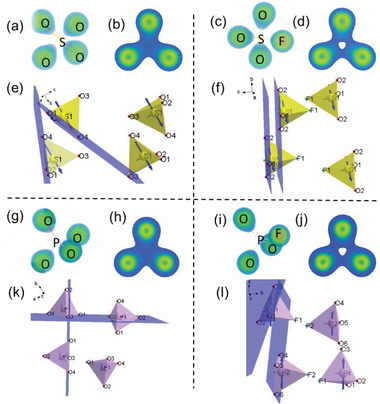
Total electron density of a) (SO_4_)^2−^ in Li_2_SO_4_, c) (SO_3_F)^−^ in LiSO_3_F, g) (PO_4_)^3−^ in Na_3_PO_4_, and i) (PO_3_F)^2−^ in Na_2_PO_3_F. And b,d,h,j) are the corresponding total electron density of the section composed by O atoms on the triangular base. Arrangement in cell of e) (SO_4_)^2−^ in Li_2_SO_4_, f) (SO_3_F)^−^ in LiSO_3_F, k) (PO_4_)^3−^ in Na_3_PO_4_, and l) (PO_3_F)^2−^ in Na_2_PO_3_F; gray and blue arrows mean the direction of *n*_min_ and *n*_max_, respectively.

According to the above calculation, one can find that fluorophosphates or fluorooxosulfates have a large birefringence compared to orthophosphates or sulfates and possess a DUV cut‐off edge. Experimental results also confirm that KSO_3_F has a larger birefringence and a DUV band gap. The determination and understanding of the structural related features of the enhanced birefringence could permit to find the corresponding criteria and to guide the design of optical materials with large birefringence. For this purpose, we analyzed these compounds from a macro perspective with Li_2_SO_4_, LiSO_3_F, Na_3_PO_4,_ and Na_2_PO_3_F as representatives. The partial density of states (PDOS) as well as the orbitals near the Fermi level show the electronic states that are related to optical properties (Figure S11, Supporting Information). In LiSO_3_F, the S, O, and F are involved in determining the band gaps. We can find that the highest occupied state levels consist of O‐2*p* and F‐2*p* and the lowest unoccupied state levels come from the hybridization between S and O, S and F. It is clear to see that the bands of Na_2_PO_3_F near the band gap are mainly determined by the O and F, *p* orbitals of the O and F sited at the valence band (VB) maximum and *p* orbitals from the P, O, and F sited at conduction band (CB) minimum. Therefore, the change in the anionic group is the main origin of the enhanced birefringence, which is also confirmed by the real‐space atom‐cutting method (Table S4, Supporting Information). In order to make the electron distribution distinguishable and comparable, a uniform density criterion was adopted (**Figure** **4**a,c,g,i). In Na_2_PO_3_F and LiSO_3_F, the electron density of the F atom is much larger than the electron densities of the O atoms, which suggests that the F atoms have a stronger binding force on electrons than the O atoms. The total electron density clearly reflects the redistribution of electrons by the form of density maps attributed to the introduction of fluorine atoms.

The symmetry of tetrahedra also changes due to the introduction of fluorine. Because of the largest electronegativity for the F atom and strong binding force on its electrons, the P/S atom is much closer to the plane constituted by the three O atoms in the tetrahedron, that is, the triangular base of the tetrahedron (Figure 4e,f,k,l). The distance between the P/S and the triangular base as well as the dihedral angle (between the triangular base and the plane composed by P/S and two O atoms on the triangular base) were measured (Table S5, Supporting Information). The distance in fluorine‐free tetrahedral group [MO_4_] (M = S, P) is about 0.48 Å while it is 0.34 Å in fluorooxo‐tetrahedral groups [MO_3_F] (M = S, P). And, the dihedral angle decreases ≈10° from the fluorine‐free tetrahedral group [MO_4_] (M = S, P) to the fluorooxo‐tetrahedral groups [MO_3_F] (M = S, P). And, the differences are also captured by the electronic densities as shown in Figure 4b,d,h,j; in Na_2_PO_3_F and LiSO_3_F, the electron clouds overlap among the three O atoms becomes smaller since the distances among them are much larger than those in Na_3_PO_4_ and Li_2_SO_4_. Obviously, the almost symmetric distribution of the O atoms around the P atom (or the S atom) in Na_3_PO_4_ (or in Li_2_SO_4_) is broken owing to the existence of the F atom. What's more, for LiSO_3_F, Li is coordinated with four oxygen atoms, whereas, Na forms NaO_5_, NaO_4_F, and NaO_5_F (Figures S12 and S13, Supporting Information); the different coordination influences the arrangement of anionic groups. As shown in Figure 4, in LiSO_3_F, the triangular bases (i.e., the O1O2O2 plane) of the (SO_3_F)^‐^ tetrahedra are parallel. And the direction of *n*_max_ is parallel to these triangular bases while the direction of *n*_min_ is vertical to these triangular bases, which means that parallel to the triangular base is the direction of maximum polarizability. In Na_2_PO_3_F, as shown in Figure 4, the triangular bases of the (PO_3_F)^2‐^ tetrahedra are not parallel and have a certain dihedral angle (*φ*), and the dihedral angle (*φ*) range from 8.67 ° to 58.97 °. Therefore, compared with Na_2_PO_3_F, the parallel arrangement of the triangular bases in LiSO_3_F is more beneficial to the superposition of the polarization anisotropy, which is accordant with the result that the birefringence increases more significantly from Li_2_SO_4_ to LiSO_3_F than that from Na_3_PO_4_ to Na_2_PO_3_F. Meanwhile, the random arrangement in KSO_3_F, RbSO_3_F, CsSO_3_F, and NH_4_SO_3_F (Figure S14, Supporting Information) is the reason for the smaller enhanced birefringence from K_2_SO_4_ (or Rb_2_SO_4_, Cs_2_SO_4_, (NH_4_)_2_SO_4_) to KSO_3_F (or RbSO_3_F, CsSO_3_F, NH_4_SO_3_F) than that from Li_2_SO_4_ to LiSO_3_F (Figure 3a; and more details are discussed in the Supporting Information). Combined with the analysis on the molecular level, it demonstrates that the introduction of the F atom results in a shift of the central atom toward the triangular base of the fluorooxo‐tetrahedron, which causes a larger polarizability anisotropy, and a parallel arrangement of the triangular base further enhances birefringence. Therefore, the overall evidence proves that the fluorooxo‐tetrahedral groups [MO_3_F] (M = S, P) are birefringence‐enhanced FMs, which will render the material a large birefringence.

## 3 Conclusion

In summary, a novel strategy of the fluorine‐free tetrahedra substituted by the fluorooxo‐tetrahedral group for the enhancement of the optical anisotropy and birefringence was proposed. From the micro perspective, the polarizability anisotropy is enhanced significantly from (PO_4_)^3−^ to (PO_3_F)^2^^−^ as well as from (SO_4_)^2−^ to (SO_3_F)^−^, while keeping large HOMO–LUMO gap. The macro optical properties of series of materials Na_3_PO_4_, Na_2_PO_3_F, Li_2_SO_4_, and LiSO_3_F were calculated and further analyzed by the REDA approach. The birefringence of Na_2_PO_3_F and LiSO_3_F is 5.5, 14.3 times those of the corresponding orthophosphate or sulfate. K_2_SO_4_, KSO_3_F, Rb_2_SO_4_, RbSO_3_F, Cs_2_SO_4_, CsSO_3_F, (NH_4_)_2_SO_4,_ and NH_4_SO_3_F were studied for their optical properties which show the similar phenomena. In addition, KSO_3_F, NH_4_SO_3_F, and the corresponding sulfates were synthesized. The millimeter crystals (10 mm × 1 mm × 1 mm for KSO_3_F, 5 mm × 1 mm × 1 mm for NH_4_SO_3_F, 3 mm × 3 mm × 1 mm for K_2_SO_4_ and (NH_4_)_2_SO_4_) were obtained to verify predicted birefringence by the polarizing microscopic method. In view of the structure, the introduction of the fluorine modulates bonding behaviors in the tetrahedron helps to reach favorably structural anisotropy. And, an optimized arrangement for the favorable micro structure can lead to a large birefringence just as in LiSO_3_F, which has the largest birefringence among ASO_3_F (A = Li, K, Rb, Cs, NH_4_), to the best of our knowledge. Furthermore, it is the first time to prove the functionality of fluorooxosulfate group as the birefringence‐enhanced FM. This study provides a feasible way to design DUV optical materials with enlarged birefringence.

## 4 Experimental Section

### Computational Details and Methods

The electronic and band structures were performed by employing CASTEP,^[^ ^40^ ^]^ a plane‐wave pseudopotential density functional theory (DFT) package, with the norm‐conserving pseudopotentials (NCPs).^[^ ^41^ ^]^ The exchange‐correlation functionals were Perdew–Burke–Emzerhof (PBE) functional within the generalized gradient approximation (GGA)^[^ ^42^ ^]^ and the HSE06 exchange‐correlation functional. The plane‐wave energy cutoff was set at 850.0 eV. Self‐consistent field (SCF) calculations were performed with a convergence criterion of 1×10^−6^ eV per atom on the total energy. The k‐points of Monkhorst–Pack grid used in the calculation of Na_3_PO_4_, Na_2_PO_3_F, Li_2_SO_4_, LiSO_3_F, K_2_SO_4_, KSO_3_F, Rb_2_SO_4_, RbSO_3_F, Cs_2_SO_4_, CsSO_3_F, (NH_4_)_2_SO_4_, and NH_4_SO_3_F were 5 × 5 × 5, 4 × 5 × 3, 3 × 4 × 3, 5 × 5 × 4, 3 × 2 × 4, 3 × 4 × 3, 3 × 2 × 4, 3 × 4 × 3, 3 × 2 × 4, 3 × 3 × 3, 3 × 2 × 4, and 2 × 4 × 3, respectively.

The orbitals and polarizability anisotropy of anionic groups were calculated using DFT implemented by the Gaussian09 package.^[^ ^43^ ^]^ B3LYP (Becke, three‐parameter, Lee‐Yang‐Parr) exchange‐correlation functional with the Lee–Yang–Parr correlation functional at the 6‐31G basis set in Gaussian being employed.

The optical anisotropy of a crystal depends on the direction of the covalent bond in the anionic groups. Therefore, birefringence is sensitive to the anisotropy of the response electron distribution, corresponding to the REDA index *ζ* = ∑_*g*_[*N*_c_ *Z*_a_Δ*ρ* ^b^/(*n*_1_ *E_o_*)]_*g*_ of the anionic groups contained in the same crystal.^[^ ^29^ ^]^ Here, *N*_c_ is the coordination number of the nearest neighbor cations to the central anion, *E*_o_ is the optical band gap, $\Delta \rho^{b}=\rho_{\max}^{b}-\rho_{\min}^{b}$, $\rho_{\max}^{b}$ and $\rho_{\max}^{b}$ are the maximum and minimum of the covalent electron density of the covalent bond on the optical principal axes of a crystal, and *n*_1_ is the minimum refractive index. Lei et al. proved the rationality of the REDA method and that the birefringence is proportional to the REDA index.^[^ ^29^ ^]^

### Synthesis

All starting reagents, K_2_SO_4_, (NH_4_)_2_SO_4_, H_2_SO_4_, KPF_6_ and NH_4_PF_6_, were commercially available with analytical grade and used without further processing. The starting materials KPF_6_, NH_4_PF_6_ (≥98 %) and H_2_SO_4_ (40 %) were purchased from Aladdin, and K_2_SO_4_, (NH_4_)_2_SO_4_, (≥ 99%) were purchased from MACKLIN. KSO_3_F was synthesized by a solvent‐free synthesis method. For KSO_3_F, a mixture of K_2_SO_4_ (0.203 g, 1mmol) and KPF_6_ (0.179 g, 1mmol) was fully ground, and 0.5 mL H_2_SO_4_ was sealed in a 23 mL Teflon autoclave and heated at 200 °C for 4 days. Then, the Teflon autoclave was slowly cooled to ambient temperature at the rate of 10 °C h^−1^. For NH_4_SO_3_F, a mixture of (NH_4_)_2_SO_4_ (0.132 g, 1mmol) and NH_4_PF_6_ (0.163 g, 1mmol) was fully ground, and 0.5 mL H_2_SO_4_ was sealed in a 23 mL Teflon autoclave and heated at 200 °C for 4 days. Then, the Teflon autoclave was slowly cooled to ambient temperature at the rate of 10 °C h^−1^. The products were washed with deionized water and dried in air. Millimeter‐sized strip‐shaped colorless transparent crystals of KSO_3_F and NH_4_SO_3_F were obtained (Figure 3e; and Figure S7a, Supporting Information). The K_2_SO_4_ and (NH_4_)_2_SO_4_ crystals were grown using the water solution method. K_2_SO_4_ (2 g) and (NH_4_)_2_SO_4_ (3 g) were dissolved in 20 and 6 mL deionized water, respectively. The solution was stirred until it became clear and evaporated at room temperature. After several weeks, colorless and millimeter‐sized single crystals of K_2_SO_4_ and (NH_4_)_2_SO_4_ were obtained (Figures S8 and S9, Supporting Information).

### Characterization

Single crystal diffraction data was collected on a Bruker SMART APEX II CCD diffractometer using monochromatic Mo K*α* radiation at 296 and 150 K and integrated with the SAINT program.^[^ ^44^ ^]^ The initial crystal structures were solved by direct methods and then refined with anisotropic displacement parameters for all atoms using the SHELXTL program package.^[^ ^45^ ^]^ The structures were verified by PLATON and no higher symmetry elements were found.^[^ ^46^ ^]^ Crystal data and structure refinement information are given in Table S1, Supporting Information. The final refined atomic positions and isotropic thermal parameters are summarized in Table S2, Supporting Information. Selected bond distances (Å) and angles (degrees) are listed in Table S3, Supporting Information. There are disorders of O/F both at 296 and 150 K (more details in the Supporting Information). The cell parameters are *a* = 8.631(5) Å, *b* = 5.857(3) Å, and *c* = 7.341(4) Å at 296 K and the *a*‐axis has a shrink at 150 K, which is discussed in detail in the Supporting Information.

Infrared spectroscopy was carried out on a Shimadzu IR Affinity‐1 Fourier transform infrared spectrometer in the 400–4000 cm^‐1^ range.

Crystals ground into a powder were characterized by NMR, which was carried out with a Bruker Avance III 500 WB (11.75 T) spectrometer operating at a frequency of 470.96 MHz for ^19^F. A commercial DVT quadruple resonance H/F/X/Y 2.5 mm CP/MAS probe was used with a spinning frequency of 30.0 kHz. Solid‐state ^19^F MAS NMR spectra were recorded with a single pulse excitation using a 90 degree pulse width of 1.9 us (pi/2) and a recycle delay of 5 s to obtain quantitative results. There was no fluorine background from the H/F/X/Y probehead. ^19^F chemical shifts were determined using a solid external reference, poly (tetrafluoroethylene) (PTFE). The CF2 groups of PTFE resonated at −122 ppm relative to tetramethylsilane (TMS).

Transmission measurement from 190 to 1600 nm was performed on a Shimadzu Solid Spec‐3700DUV spectrophotometer by using a transparent unpolished crystal with a thickness of ≈1 mm.

The birefringence was characterized by using the polarizing microscope equipped (ZEISS Axio Scope. A1) with Berek compensator. The wavelength of the light source was 546 nm. Owing to the clear boundary lines of the first‐, second‐, and third‐order interference color, the relative error was small enough. Before the scanning, the small and transparent lamellar crystal were chosen to measure, in order to improve the accuracy of the birefringence. The formula for calculating the birefringence is listed below,

(1) $$R=\left|N_{e}-N_{o}\right|\times T=\Delta n\times T$$

Here, *R* represents the optical path difference, Δ*n* means the birefringence, and *T* denotes the thickness of the crystal.^[^ ^39^ ^]^

## Conflict of Interest

The authors declare no conflict of interest.

## Supporting information

Supporting Information

## Data Availability

Research data are not shared.
