# Supplementary material for: Fluorine‐Driven Enhancement of Birefringence in the Fluorooxosulfate: A Deep Evaluation from a Joint Experimental and Computational Study
Source: Adv Sci (Weinh). 2021 Jun 4;8(15):2003594. doi: 10.1002/advs.202003594 (PMC8336506; doi:10.1002/advs.202003594)
Supplement: Supplementary file 1 — Supporting Information [file ADVS-8-2003594-s001.pdf]

© 2021 Wiley-VCH GmbH

ADVANCED SCIENCE
Open Access

Supporting Information

for *Adv. Sci.,* DOI: 10.1002/advs.202003594

Fluorine-Driven Enhancement of Birefringence in the Fluorooxosulfate： A Deep Evaluation from a Joint Experimental and Computational Study

Wenqi Jin, Wenyao Zhang, Abudukadi Tudi, Liying Wang, Xin Zhou, Zhihua Yang, Shilie Pan**

Copyright Wiley-VCH GmbHWeinheim, Germany, 2021.

Supporting Information

Fluorine-Driven Enhancement of Birefringence in the Fluorooxosulfate：A Deep Evaluation from a Joint Experimental and Computational Study

WenqiJin,Wenyao Zhang, AbudukadiTudi,Liying Wang, Xin Zhou, Zhihua Yang,Shilie Pan**

Structure refinement

In this work, the crystal data were re-collected at293 and 150 K. We refined the structure, explained and proved the disorders of O/F. The final refined site occupation factors of O1/F1, O2/F2and O3/F3 are 0.53/0.47,0.61/0.39 and 0.93/0.07 at293 K, and are 0.38/0.62, 0.67/0.33 and 0.98/0.02 at 150 K. Furthermore, the structure was checked for possible higher symmetry using the ADDSYM algorithm from the program PLATON and suggested space groupas *Pnma*. Therefore, the structure KSO_3F was solved in the space group *Pnma* with disorder, and a satisfying structure model with very low convergence factor (R_1 = 0.0167) can be obtained.Furthermore, for the temperature decreased the *a*-axis shrinks dramatically stronger than the other two,this may be due to the anisotropicexpansion of the crystal. According to the final refinedsite occupation factors of O/F, the site occupation factor of O1 decreases from 0.53 to 0.38 with the temperature decreasing. As shown in Figure S15, we find that the bond distance of S-O(1)/F(1) in theSO_3F tetrahedron increases from 1.488 to 1.527 Å due to

the site occupation factor of F1 increasing from 0.47 to 0.62.The bond distance of 1.527Å is closer to the previous reported S-F bond distance and is larger than S-O bonds, which further confirmsthe presence of S-F bonds.Furthermore, the arrangement direction of S-O(1)/F(1) is along the *a*-axis. The distance between two $(SO_3F)^-$ tetrahedra has decreased from 4.397 to 4.3045 Å with temperature decreased, however, it just decreased from 5.857 to 5.854 Åalong the *b*-axis. So, we believe that the temperature is a key factor for the site occupation factor of O1/F1, and further influences the cell parameters.

NMR spectroscopy

For further to provide sufficient evidence, theNMRspectroscopy was applied.According to the ^{19}F spectroscopy, we find that thedominant signal at 38.7 ppm may be assigned to F nearest to K. The signals in the chemical-shift regionfrom -66.5to -74.5 ppmmay be assigned to ^{19}F in tetrahedron$(SO_3F)^-$with various nearest neighbor substituents.It is clear that such chemical-shift range spectrum consists of two types of signals which are characterized by narrow and broad peaks, respectively (see the insert in Figure S3).These broad signals are due to the disorder produced by the distribution of F and O atoms in the same crystallographic site, which is consistent with the previous reported.[1] As we allknow, the chemical shifts of ^{19}F are very sensitive to the coordination environment of Fatom. The occupation factors of O1/F1,O2/F2and O3/F3are different, and the corresponding chemical shifts of ^{19}F are different, these different ^{19}F-NMR signals overlap to produce the broadsignal.

Detailed discussion about the calculation of the KSO_3F and $RbSO_3F$

In the process of calculation, virtual crystal approximation (VCA) method[2] was adopted to model the disorderd structure. We chose two model structures (named KSO_3F (model I) and KSO_3F (model II)) with the occupation of O2 (or O1) being 100% while the F1 (or F2) being 100%, and the occupation of O3 being 100%. And then the BFGS[3] geometry optimization was performed for the two structure models, and the geometry optimization completed successfully. The calculated band gap and birefringence for the two models are listed in Table S8. The two models have different band gap (6.86 eV for model I and 6.92 eV for model II) and birefringence (0.024 for model I and 0.034 for model II). So from the two models, we find that the fluorine atoms can affect the birefringence. In the structure of KSO_3F obtained from experiment, the final refined site occupation factors of O1/F1, O2/F2and O3/F3 are0.53/0.47,0.61/0.39 and 0.93/0.07, respectively. Therefore, the model I is more closer to the experimental structure. What's more, the calculated birefringence of KSO_3F (model I) is more consistent with the experimental birefringence of KSO_3F, so we adopted the structure model (I) for the further analysis. The same strategy was adopted for $RbSO_3F$.This strategy has been used in the previous work.[4]

Detailed discussion about the birefringences of ASO_3F (A = Li, K, Rb, Cs)

The partial density of states (PDOS)as well as the orbitals (taking the $LiSO_3F$ as example) near the Fermi level show that the $(SO_3F)^-$ is the main origin of the enhanced birefringence, which is also confirmed by the real-space atom-cutting method (Table

S4).For the lithium fluorooxosulfate, Li is coordinated with four oxygen atoms, whereas, K, Rb, Cs form KO_7F, RbO_7F, CsO_8F (Figure S12), thedifferent coordination influences the arrangement of anionic groups.As shown in Figure S14, in the lithium fluorooxosulfate, the triangular bases (i.e. the O1O2O2 plane) of the $(SO_3F)^-$tetrahedra are parallel.And the direction of $n_{max}$is parallel to these triangular bases while the direction of $n_{min}$is vertical to these triangular bases, which means that parallel to the triangular base is the direction of maximum polarizability. In other alkali metal fluorooxosulfates, as shown in Figure S14, the triangular bases of the $(SO_3F)^-$tetrahedra are not parallel and have a certain dihedral angle (φ).The dihedral angles (φ) arelisted in Table S6, from which we can see that the structure with a smaller dihedral angel has a larger birefringence generally. What's more, the density of birefringence-active groups also influences the birefringence. Therefore, considering the influence of the dihedral angle (φ) between the triangular bases in thestructure and the density (ρ) of anionic group, we defined a geometric factor $N = \rho \cdot \cos\varphi$.From Figure S16, it can be seen that the birefringence-active groups with optimized arrangement and high-density are beneficial to the enhancement of the birefringence. Herein, $LiSO_3F$ has the largest N value, corresponding to the largest birefringence.

Table S1. Crystallographic data for KSO_3F.

Empiricalformula	KSO_3F
Formula weight	138.16

Temperature (K)	296(2)	150(2)
Wavelength (Å)	0.71073	
Crystal system	Orthorhombic	
Space group	*Pnma*	
a / Å	8.631(5)	8.4604(17)
b / Å	5.857(3)	5.8542(11)
c / Å	7.341(4)	7.2884(13)
α /°	90	
β /°	90	
γ/°	90	
Z, ρ_{calcd}/ g·cm^{-3}	4, 2.473	4,2.542
Volume /Å^3	371.1(4)	360.99(12)
Reflections collected / unique	2145 / 472 [R_{int} = 0.0228]	4424 / 456[R_{int} = 0.0367]
Completeness (%)	100	99.7
Goodness-of-fit on F^2	1.092	1.158
Final R indices [I>2sigma(I)] [a]	R_1 = 0.0217, wR_2 = 0.0543	R_1 = 0.0167, wR_2 = 0.0466
R indices (all data)[a]	R_1 = 0.0243, wR_2 = 0.0553	R_1 = 0.0176, wR_2 = 0.0469
Extinction coefficient	0.052(5)	0.042(5)
Largest diff. peak and hole (e·Å^{-3})	0.282 and -0.280	0.198 and -0.433

[a]$R_1 = \Sigma||F_o| - |F_c||/\Sigma|F_o|$ and $wR_2 = [\Sigma w(F_o^2 - F_c^2)^2 / \Sigma wF_o^4]^{1/2}$ for $F_o^2 > 2\sigma(F_o^2)$

Table S2. Atomic coordinates ($\times10^4$) and equivalent isotropic displacement parameters ($Å^2\times 10^3$) forKSO_3F. U_{eq} is defined as one-third of the trace of the orthogonalizedU_{ij} tensor.

Atom	S.O.F	*x*	*y*	*z*	U_{eq}
		296 K			
K(1)		-3240(1)	2500	6603(1)	35(1)
S(1)		692(1)	2500	3073(1)	30(1)
O(1)	0.53	1940(2)	2500	4473(2)	48(1)
O(2)	0.61	-782(2)	2500	4099(3)	55(1)
O(3)	0.93	810(2)	457(2)	2016(2)	41(1)
F(1)	0.47	1940(2)	2500	4473(2)	48(1)
F(2)	0.39	-782(2)	2500	4099(3)	55(1)
F(3)	0.07	810(2)	457(2)	2016(2)	41(1)
		150 K			
K(1)		8257(1)	7500	6597(1)	19(1)
S(1)		4311(1)	7500	3046(1)	18(1)
O(1)	0.38	2982(2)	7500	4464(2)	25(1)
O(2)	0.67	5770(2)	7500	4158(2)	29(1)
O(3)	0.98	4171(1)	5438(2)	2007(1)	22(1)
F(1)	0.62	2982(2)	7500	4464(2)	25(1)
F(2)	0.33	5770(2)	7500	4158(2)	29(1)
F(3)	0.02	4171(1)	5438(2)	2007(1)	22(1)

Table S3. Selected bond distances (Å) and angles (deg.) forKSO_3F.

296 K		150 K	
K(1)-O(2)	2.807(2)	K(1)-O(2)	2.7546(15)
K(1)-O(3)#1	2.8302(18)	K(1)-O(3)#1	2.7895(11)
K(1)-O(3)#2	2.8302(18)	K(1)-O(3)#2	2.7895(11)
K(1)-O(1)#3	2.885(2)	K(1)-O(3)#3	2.8661(11)
K(1)-O(3)#4	2.9031(18)	K(1)-O(3)#4	2.8661(11)
K(1)-O(3)#5	2.9031(18)	K(1)-O(1)#5	2.8805(14)
K(1)-O(3)#6	3.027(2)	K(1)-O(3)#6	2.9927(11)
K(1)-O(3)#7	3.027(2)	K(1)-O(3)#7	2.9927(11)
K(1)-O(1)#8	3.2338(18)	K(1)-O(1)#3	3.2037(8)
K(1)-O(1)#5	3.2338(18)	K(1)-O(1)#8	3.2037(8)
K(1)-S(1)#6	3.554(2)	S(1)-O(3)/F(3)#9	1.4298(10)
S(1)-O(3)	1.4296(14)	S(1)-O(3)/F(3)	1.4299(10)
S(1)-O(3)#9	1.4296(14)	S(1)-O(2)/F(2)	1.4760(14)
S(1)-O(2)	1.4784(19)	S(1)-O(1)/F(1)	1.5274(13)
S(1)-O(1)	1.4889(18)	O(3)#9-S(1)-O(3)	115.18(9)
O(3)-S(1)-O(3)#9	113.62(12)	O(3)#9-S(1)-O(2)	111.09(5)
O(3)-S(1)-O(2)	109.73(7)	O(3)-S(1)-O(2)	111.09(5)
O(3)#9-S(1)-O(2)	109.73(7)	O(3)#9-S(1)-O(1)	107.30(5)
O(3)-S(1)-O(1)	108.87(7)	O(3)-S(1)-O(1)	107.30(5)
O(3)#9-S(1)-O(1)	108.87(7)	O(2)-S(1)-O(1)	104.14(8)

Symmetry transformations used to generate equivalent atoms:

#1 -x-1/2,-y,z+1/2	#2 -x-1/2,y+1/2,z+1/2	#3 x-1/2,y,-z+3/2
#4 -x,y+1/2,-z+1	#5 -x,-y,-z+1	#6 x-1/2,y,-z+1/2
#7 x-1/2,-y+1/2,-z+1/2	#8 -x,-y+1,-z+1	#9 x,-y+1/2,z

Table S4. Results of the real-space atom-cutting for $LiSO_3F$ and Na_2PO_3F.

Compounds	Δ*n* (@1064nm)	Cut Li/Cut Na	Cut SO_3F/Cut PO_3F
$LiSO_3F$	0.054	0.054	0.0001
Na_2PO_3F	0.022	0.022	0.0006

Table S5. Distance between P/S atom and the triangular base and the dihedral angle between PO/SO plane and the triangular base.

Coumpounds	Atom-Plane	Distance (Å)	Plane-Plane	Angle (°)
Na_3PO_4	P1-O1O3O4	0.4887	P1O1O3-O1O3O4	33.38
			P1O1O4-O1O3O4	34.25
			P1O3O4-O1O3O4	33.97
Na_2PO_3F	P1-O1O2O3	0.3403	P1O1O2-O1O2O3	25.09
			P1O1O3-O1O2O3	24.97
			P1O2O3-O1O2O3	24.81
	P2-O4O5O6	0.3528	P2O4O5-O4O5O6	25.79
			P2O4O6-O4O5O6	25.60
			P2O5O6-O4O5O6	26.10
Li_2SO_4	S1-O1O2O4	0.4873	S1O1O2-O1O2O4	34.39
			S1O1O4-O1O2O4	34.61
			S1O2O4-O1O2O4	35.14
$LiSO_3F$	S1-O1O2O2	0.3365	S1O1O2-O1O2O2	25.03
			S1O1O2-O1O2O2	25.03
			S1O2O2-O1O2O2	26.37

Table S6. Dihedral angle (*φ*) between the triangular bases.

Compounds	*φ* (°)
$LiSO_3F$	0.00
KSO_3F	80.46
$RbSO_3F$	79.61
$CsSO_3F$	61.16

Table S7. Data of cos*φ*, *ρ*, *N* value and birefringence.

Compounds	cos*φ*	*ρ* (×10^{-3}·Å^{-3})	*N* (×10^{-3})	Δ*n*
$LiSO_3F$	1	9.492	9.49	0.057
KSO_3F	0.1657	10.074	1.67	0.024
$RbSO_3F$	0.1804	9.023	1.63	0.022
$CsSO_3F$	0.4824	8.046	3.88	0.031

Table S8. Bandgap and birefringence for the models of KSO_3F and $RbSO_3F$.

Structures	E_g (eV) [HSE06]	Δn
KSO_3F (model I)	6.86	0.024
KSO_3F (model II)	6.92	0.038
$RbSO_3F$ (model I)	6.75	0.022
$RbSO_3F$ (model II)	6.80	0.034

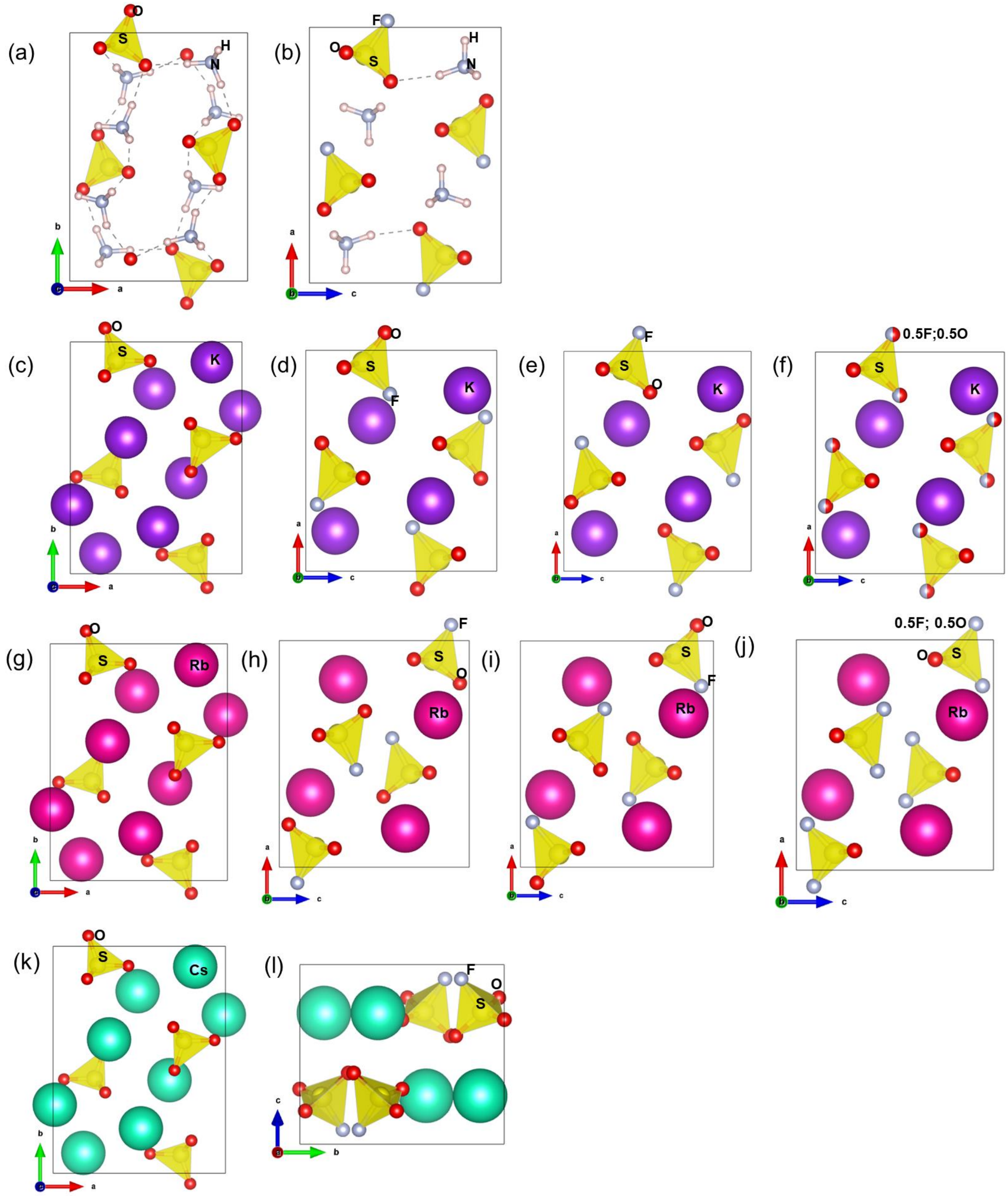

Figure S1. Optimized crystal structures of (a) $(NH_4)_2SO_4$, (b) NH_4SO_3F,(c) K_2SO_4, (d) KSO_3F (model I), (e) KSO_3F (model II), (g) Rb_2SO_4, (h) $RbSO_3F$ (model I), (i) $RbSO_3F$ (model II), (k) Cs_2SO_4, (l) $CsSO_3F$. Without treated and optimized crystal structure of (f) KSO_3F and (j) $RbSO_3F$.

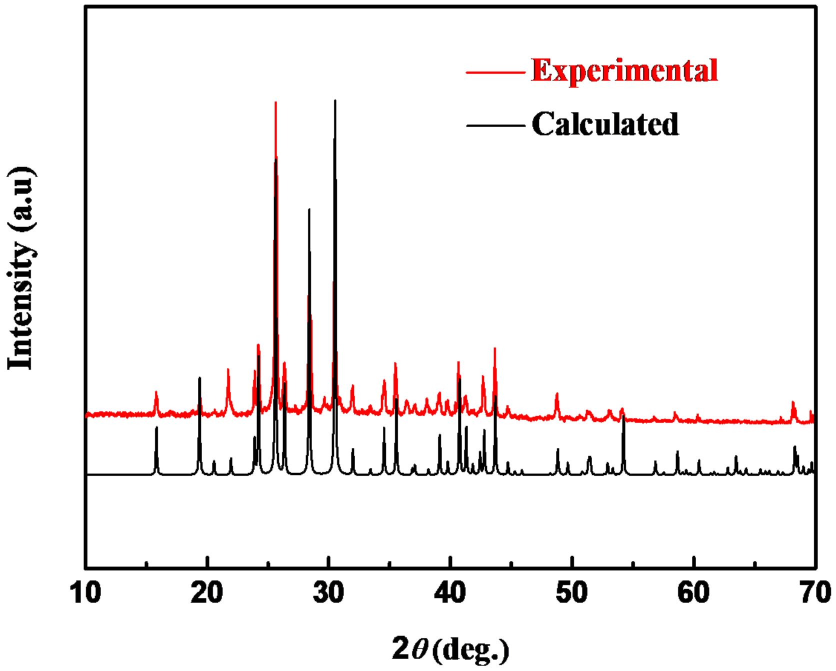

Figure S2. X-ray powder diffraction patterns of KSO_3F.

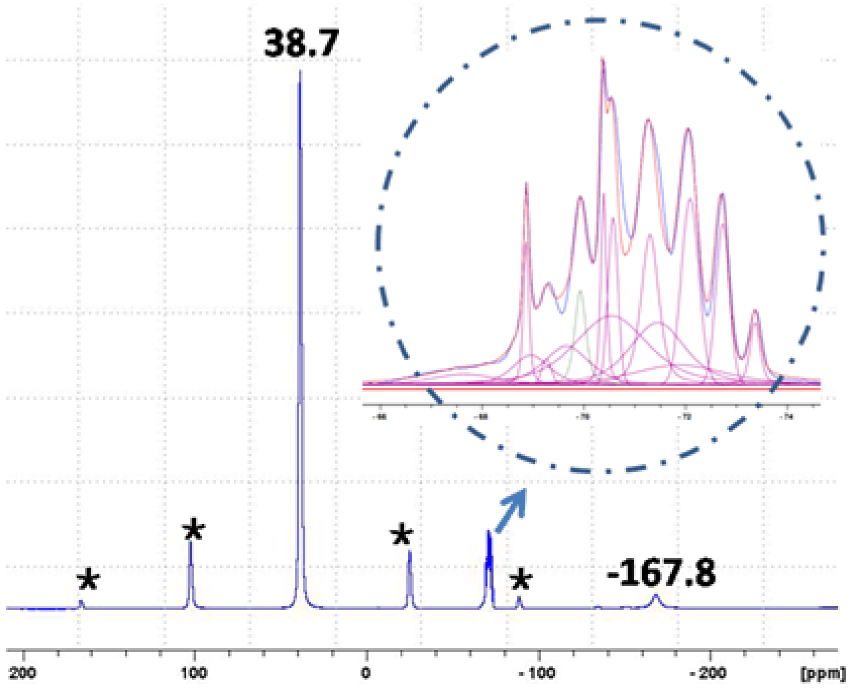

Figure S3.^{19}F MAS NMR spectrum of KSO_3F. Asterisks denotespinningsidebands.

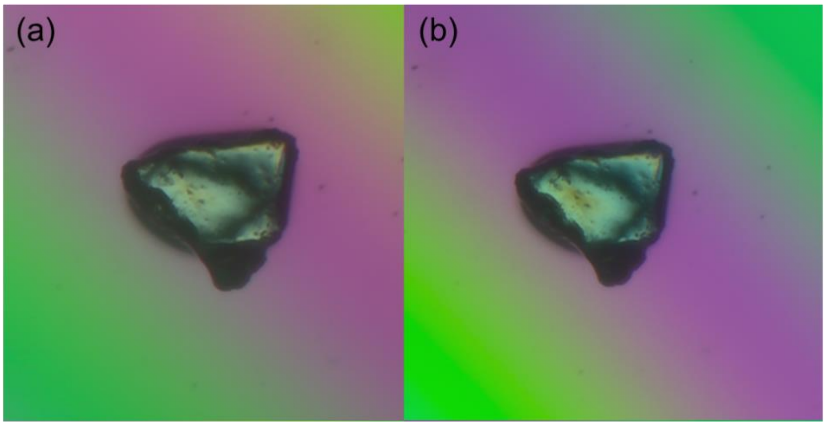

Figure S4. The KSO_3F single crystal under the polarizing microscope.

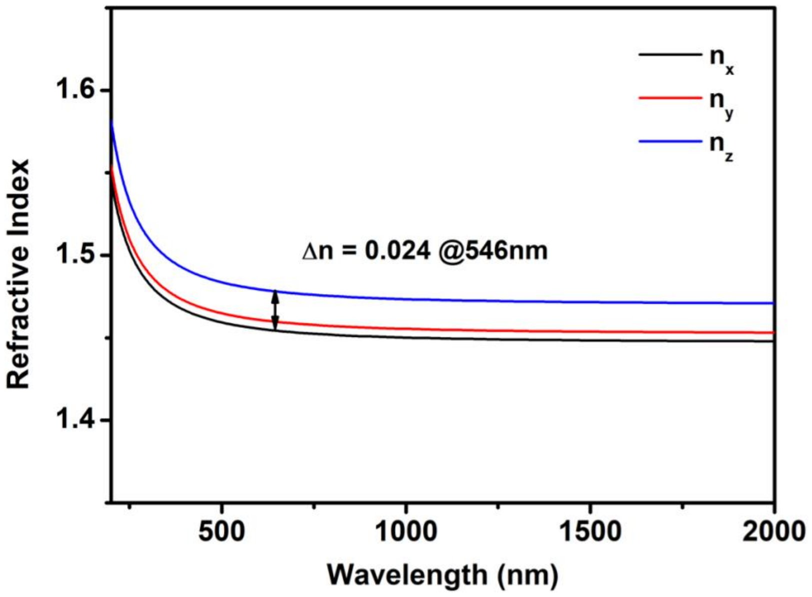

Figure S5. Calculated refractive index dispersion curves of KSO_3F.

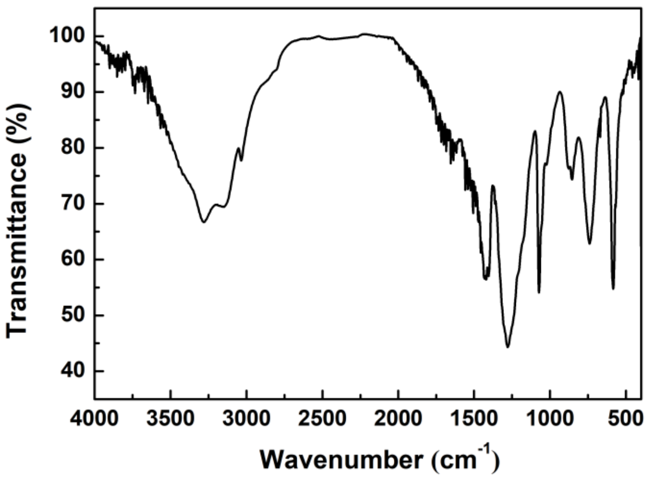

Figure S6. IR spectrum of NH_4SO_3F.The peaks at 570 and 590 cm^{-1} are attributed to O-S-F formation vibration and O-S-O formation vibration, respectively. The peak at 750 cm^{-1} is interpreted for the S-F stretching vibration. The peaks at 1080 and 1280 cm^{-1} are ascribed for S-O symmetrical stretching vibration and S-O asymmetrical stretching vibration, respectively. The strong absorptions in the 3500–2900 $cm^{–1}$ range are consistent with the NH^{4+} stretching vibration.

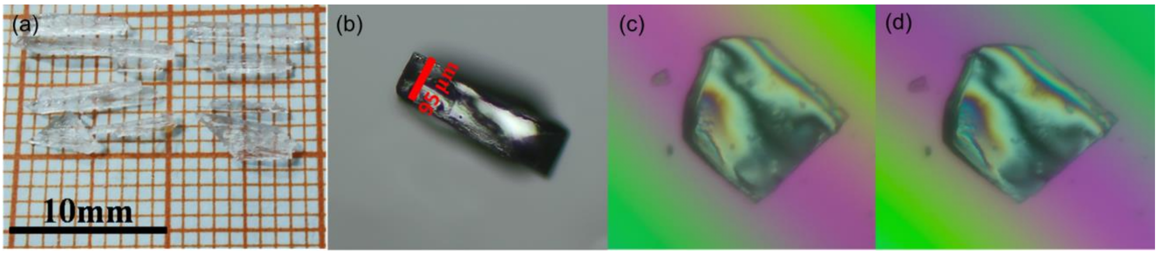

Figure S7. (a) Crystals of NH_4SO_3F. (b) The thickness of NH_4SO_3F crystal. (c), (d) The NH_4SO_3F single crystal under the polarizing microscope. The measured refractive index difference for NH_4SO_3F is 0.018 and the real birefringence should be equal or larger than 0.018.

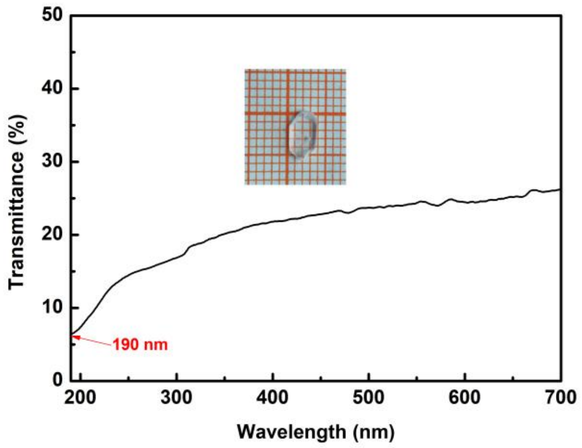

Figure S8. Transmission spectrum of K_2SO_4 with dimensions of 3 mm × 3 mm × 1 mm (inset).

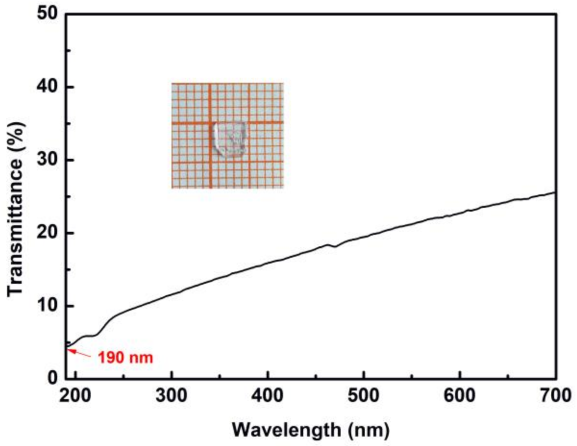

Figure S9. Transmission spectrum of $(NH_4)_2SO_4$ with dimensions of 3 mm × 3 mm × 1 mm (inset).

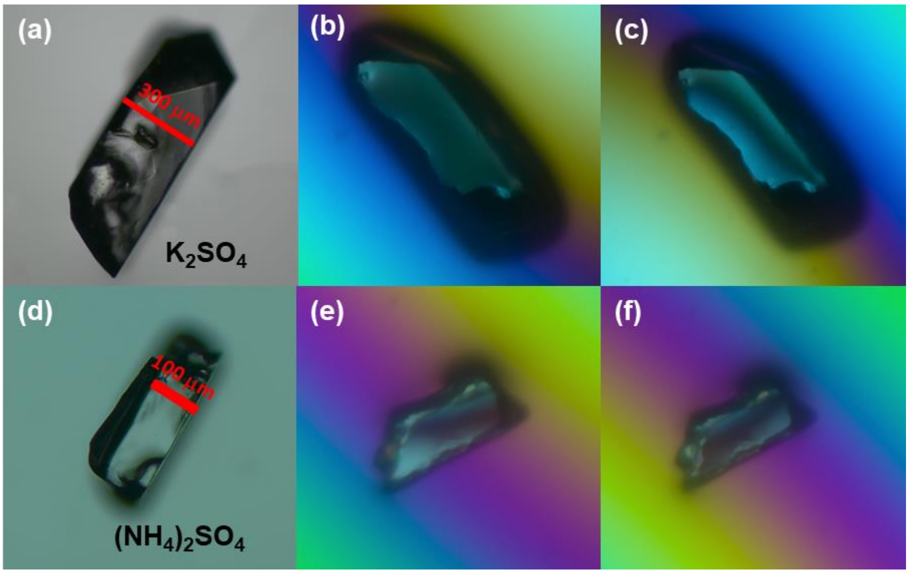

Figure S10. (a) The thickness of K_2SO_4 crystal. (b), (c) The K_2SO_4 single crystal under the polarizing microscope. (d) The thickness of $(NH_4)_2SO_4$ crystal. (e), (f) The $(NH_4)_2SO_4$ single crystal under the polarizing microscope. The measured refractive index difference for this K_2SO_4 single crystal is 0.002 and the real birefringence should be equal or larger than 0.002. The measured refractive index difference for this $(NH_4)_2SO_4$ single crystal is 0.011 and the real birefringence should be equal or larger than 0.011.

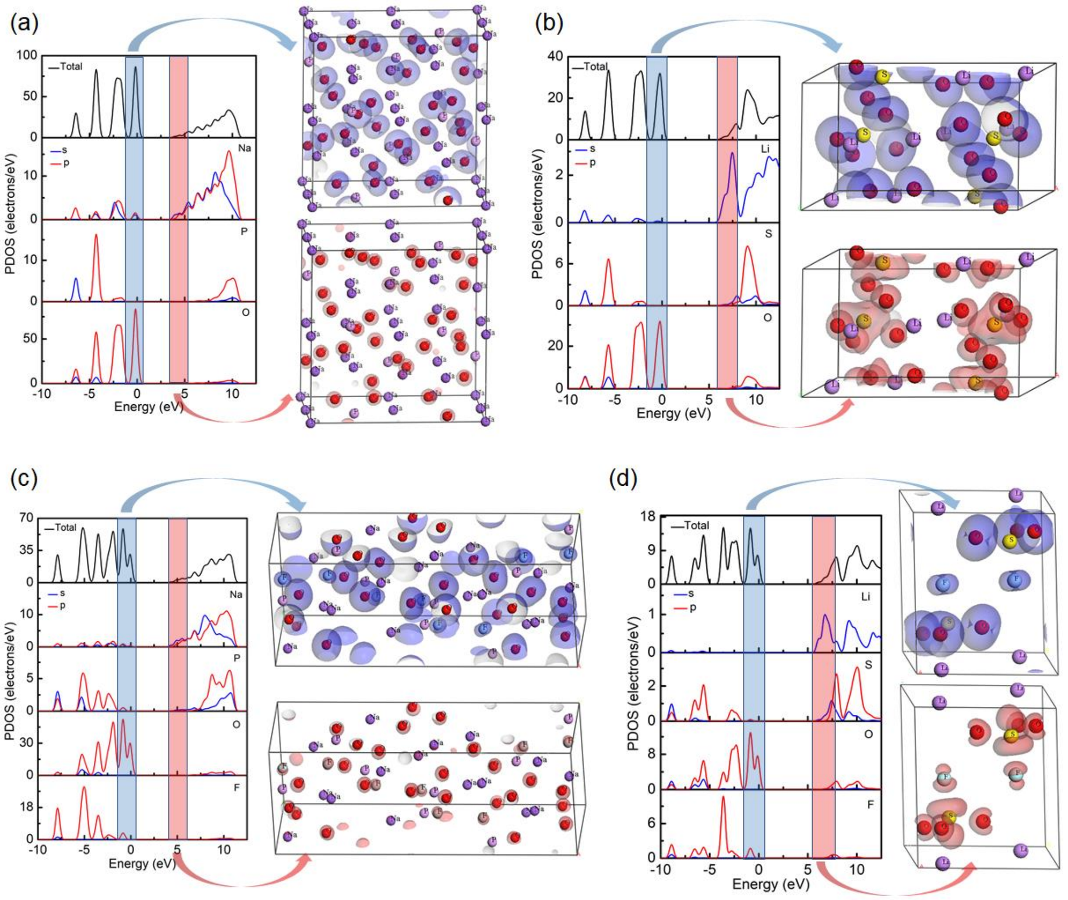

Figure S11. Partial density of states (PDOS) and the specific orbitals in the energy range near the band gaps for (a) Na_3PO_4, (b) Li_2SO_4, (c) Na_2PO_3F and (d) $LiSO_3F$.

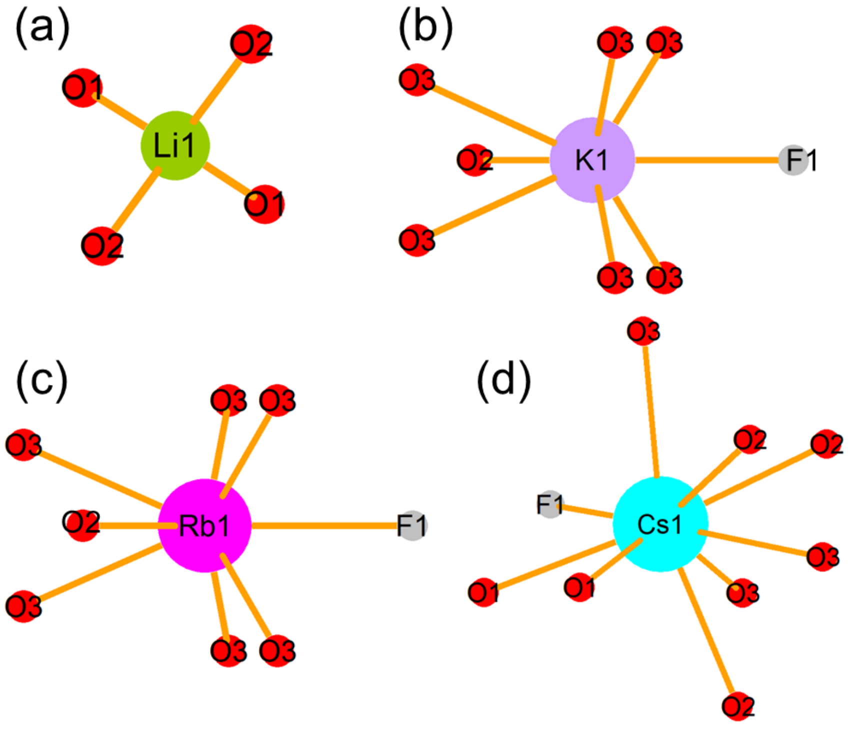

Figure S12. Coordination environment of metal cation in (a) $LiSO_3F$, (b) KSO_3F, (c) $RbSO_3F$, (d) $CsSO_3F$.

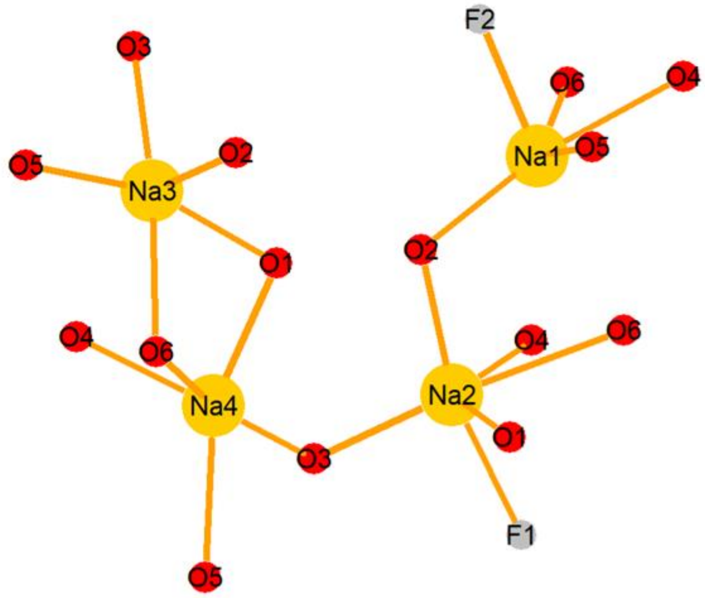

Figure S13. Coordination environment of metal cation in Na_2PO_3F.

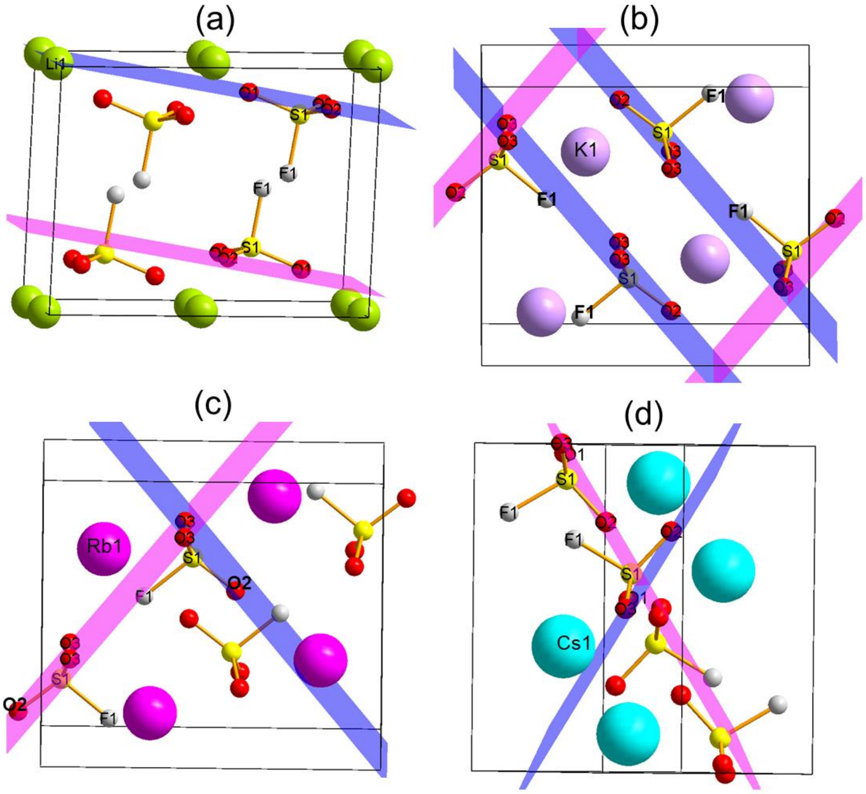

Figure S14. Planes of the triangular base in (a) $LiSO_3F$, (b) KSO_3F, (c) $RbSO_3F$, (d) $CsSO_3F$. Notably, taking KSO_3F as an example, the same planes were shown in same color.

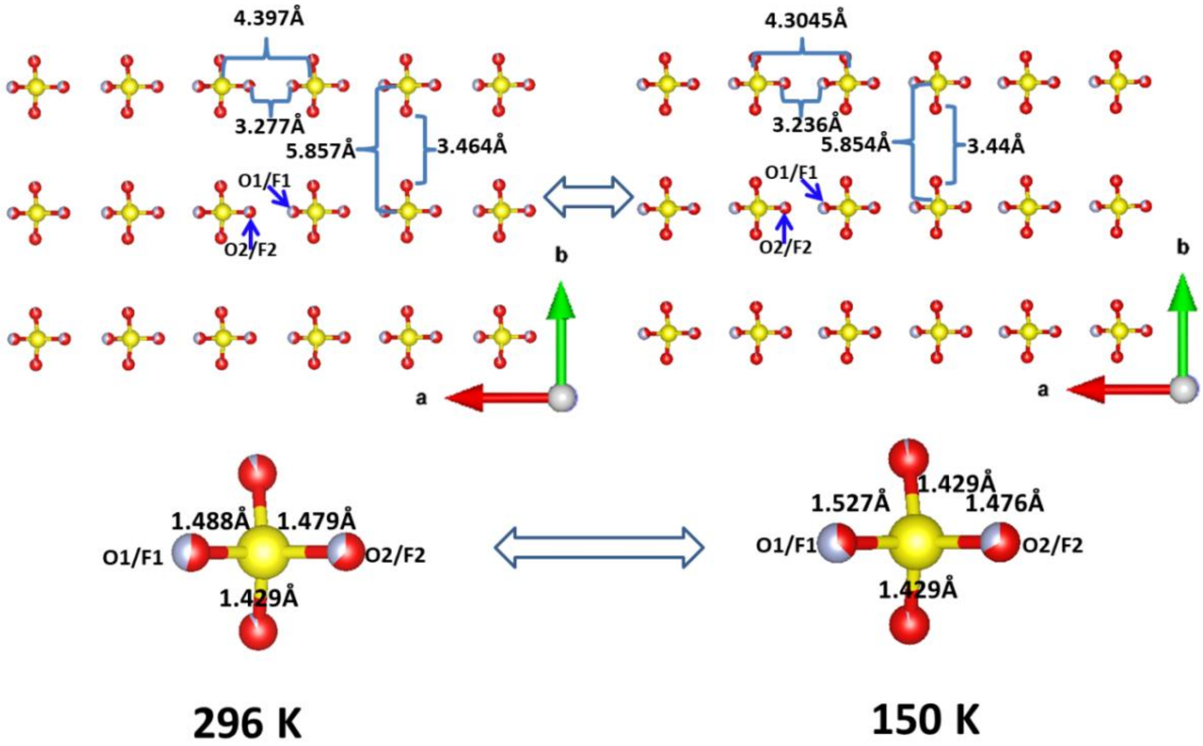

Figure S15. Crystal structures at different temperatures.

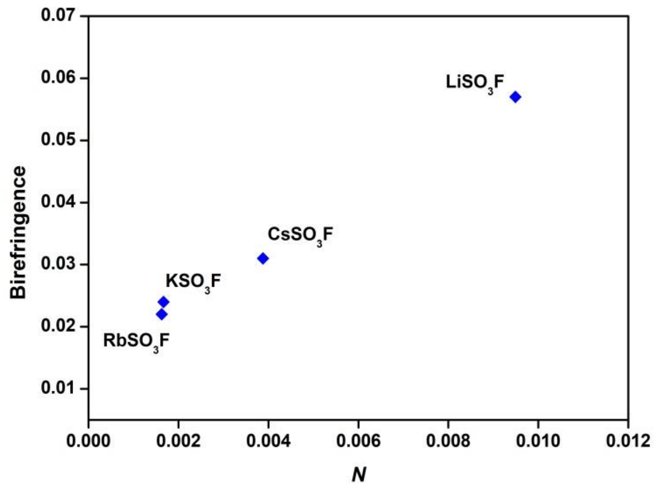

Figure S16. Birefringence and *N* value of $LiSO_3F$, KSO_3F, $RbSO_3F$, $CsSO_3F$.

References

[1] J. Dabachi, M. Body, C. Galven, F. Boucher, C. Legein, *Inorg. Chem.***2017**, *56*, 5219.

[2] L. Bellaiche, D. Vanderbilt, *Phys. Rev. B***2000**, *61*, 7877.

[3] T. H. Fischer, J. Almlof, *J. Phys. Chem.***1992**, *96*, 9768.

[4] a) M. L. Zhou, Y. Yang, Y. W. Guo, Z. S. Lin, J. Y. Yao, Y. C. Wu, C. T. Chen, *Chem. Mater.***2017**, *29*, 7993; b) X. Yan, S. Y. Luo, Z. S. Lin, Y. C. Yue, X. Y. Wang, L. J. Liu, C. T. Chen, *J. Mater. Chem. C***2013**, *1*,3616; c) S. J. Han, C. M. Huang, A. Tudi, S. S. Hu, Z. H. Yang, S. L. Pan, *Chem. Eur. J.***2019**, *25*, 11614.
